# Provision of palliative care for chronic heart failure inpatients: how much do we need?

**DOI:** 10.1186/1472-684X-8-8

**Published:** 2009-06-29

**Authors:** Richard Harding, Teresa Beynon, Fiona Hodson, Elaine Coady, Mark Kinirons, Lucy Selman, Irene Higginson

**Affiliations:** 1 Department of Palliative Care, Policy and Rehabilitation, King's College London, London, UK; 2 Department of Palliative Medicine, Guy's and St Thomas' NHS Foundation Trust, London, UK; 3 Heart Failure Service, Guy's and St Thomas' NHS Foundation Trust, London, UK; 4 Elderly Medicine, Guy's and St Thomas' NHS Foundation Trust, London, UK

## Abstract

**Background:**

Clinical guidance recommends early CHF palliative care intervention, but the magnitude of need is unknown and evidence-based referral criteria absent.

This study aimed to: 1) Measure point prevalence of inpatients appropriate for palliative care. 2) Identify patient characteristics associated with palliative care appropriateness. 3) Propose evidence-based clinical referral criteria.

**Methods:**

Census: all adult medical inpatient files in a UK tertiary teaching hospital were reviewed, identifying patients with CHF as a reason for current admission, using NYHA stage 3/4 classification, cross referenced with existing ECHO data. Each CHF patient was classified according to appropriateness for palliative care against a definition of unresolved pain and/or symptoms and/or psychosocial problems 7 days post admission.

**Results:**

Three hundred and sixty-five patient files were reviewed, and 28 clinically identified as having CHF. Of these, 11 had confirmed unpreserved ejection fraction,16 of the 28 patients were appropriate for palliative care. Of the total inpatient population reviewed, 10 (2.7%) had both confirmed ejection fraction ≤45%, and were appropriate for palliative care. Of the 17 clinically-identified CHF patients with no recorded evidence of ejection fraction ≤45%, 5 (29.4%) were still appropriate for palliative care. A total of 4.4% of the reviewed inpatient population had a clinical diagnosis of CHF and were appropriate for palliative care.

**Conclusion:**

CHF patients with ejection fraction >45% also require palliative care. Our conservative criteria suggest a point prevalence of 2.7% of patients having both ejection fraction ≤45% and palliative care needs, although this may be a conservative estimate due to the file review methodology to identify unresolved palliative care problems. It is important to note that the point prevalence of patients with clinical diagnosis and palliative care needs was 4.4% of the population. We present evidence-based referral criteria from the larger multi methods study.

## Background

End stage Chronic Heart Failure (CHF) is associated with high pain and symptom burden (e.g. 60–88% breathlessness, 42–82% fatigue, 41–77% pain, 17–48% nausea)[1,2] and mortality rates are poor among those newly diagnosed with heart failure (70% survival at 6 months and 57% at 18 months). [3] The majority of admissions (72%) are unplanned, [4] and around one half of CHF patients die suddenly rather than dying of progressive heart failure. [5] As new treatments extend the unpredictable chronic disease phase,[6] both the incidence and prevalence of chronic heart failure (CHF) are predicted to rise substantially. [7]

Patients with CHF should be treated throughout the entire disease trajectory,[8] and the National Institute for Clinical Excellence (NICE) CHF clinical guidance requires that 'The palliative needs of patients and carers should be identified, assessed and managed at the earliest opportunity '. [9]The aim of palliative care is to clinically manage complex (and often apparently refractory) symptoms, provide psycho-social support to the patient and their family, to improve quality of remaining life, achieve the best possible death, and should be available from the point of diagnosis through to the end of life. [10] However, there is currently no data to model the magnitude of palliative care provision required to meet guidance requirements.

This paper reports census data as part of a larger mixed methods investigation to develop an evidence-based CHF palliative care service. [10-12] The present phase of investigation aimed firstly to inform resource allocation and service planning, by quantifying the number of CHF patients with potential palliative care needs through a one-day census of adult inpatient notes at a central London teaching hospital. The second aim was to identify patient characteristics associated with CHF-related palliative care appropriateness to inform referral criteria. The third aim was to generate evidence based referral criteria to specialist palliative care using the findings of the multi methods study data, [10-12] to address the challenges in prognostication and the uncertainty within cardiology as to when palliative care should be initiated and for which needs.

## Methods

### Design

This study utilised a one-day census method to measure the number of inpatients with CHF and those appropriate for palliative care.

### Setting

The census was conducted at a large, tertiary central London (UK) teaching hospital.

### Inclusion/exclusion criteria

Inclusion criteria for file review were all adult inpatient files on general medical, vascular surgical and care of the elderly wards plus the acute admissions observation room. Exclusion criteria were those in Accident and Emergency, the Surgical, Obstetric and Gynaecological, and Paediatric wards.

### Definitions

CHF was firstly identified clinically as being recorded in patient notes as a significant reason for admission, according to the New York Heart Association (NYHA) classification as Class III (marked limitation of activity; comfortable only at rest) or IV (should be at complete rest, confined to bed or chair; any physical activity brings on discomfort and symptoms occur at rest). In addition to this clinical definition of CHF, those clinically identified as having CHF had their medical records reviewed for most recent echocardiogram data (ECHO).

The operational definition of being appropriate for palliative care in this study was recorded unresolved pain/symptoms and/or complex psychosocial needs seven days post-admission. Therefore any patient still present on the ward seven days after admission and present at the point of review could potentially have been included.

### Procedure

First, each patient was reviewed and coded by their ward medical staff according to whether they had a recorded clinical diagnosis of CHF as a significant reason for their current admission. Second, those with a clinical diagnosis of CHF had demographic and clinical file data extracted (i.e. left ventricular ejection fraction/ECHO, number of previous admissions, pain, symptoms, do not resuscitate and psychosocial problems). Third, those with clinical diagnosis of CHF were coded according to whether they were appropriate for palliative care using the above definition.

The census was conducted on one day. Appropriateness for palliative care was jointly reviewed according to classification by the completing medical staff and a palliative care consultant.

Ethical approval was granted for the full mixed-methods study protocol under a single application to St Thomas' Hospital Research Ethics Committee (approval ref 05/Q0702/5).

### Analysis

Data were extracted on the wards into piloted data extraction sheets, and subsequently entered into SPSS for analysis.

Descriptive data on the CHF patients were produced, and point prevalence of both CHF and being appropriate for palliative care calculated from the entire patient population reviewed in the census. Data were analysed exploring both those with and without evidence of ejection fraction ≤45%. Classification as appropriate/inappropriate for palliative care was further explored, comparing number of previous admissions, and multiprofessional staff input (each using parametric comparison of means) and presence of "do not resuscitate" orders on file (chi square).

## Results

### Sample characteristics

**Figure 1:**
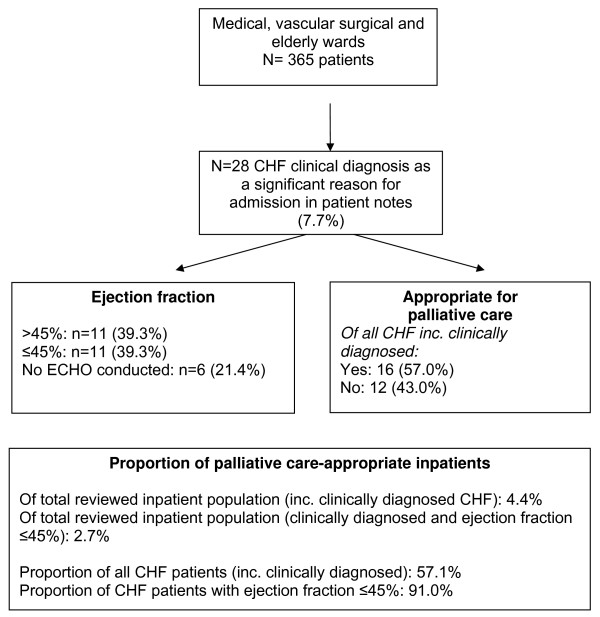
**Flow chart: sample description**.

Of 365 reviewed beds (on 14 wards and three High Dependency Units), 28 patients were clinically identified as having CHF. The number of multi-professional inpatient staff being seen by the whole sample of 28 patients was as follows: Physiotherapist n = 14, Occupational Therapist n = 10, Dietician n = 6, Social Worker n = 5, Speech and Language Therapist n = 2, Discharge Co-ordinator n = 2, Pain Team n = 1. The mean number of professionals was 1.6. The data flow chart is presented in figure 1. Patient characteristics are presented in Table 1.

**Table 1:** Patient characteristics

	**All pts with clinical CHF diagnosis** **N = 28**	**Appropriate for palliative care** **N = 16**	**Not appropriate for palliative care** **N = 12**
**Mean age (standard deviation)**	78 (SD = 13.1)	76.4 (13.5)	80.1 (12.7)
**Sex**	17 (60.7%) female	6 (37.5%) female	11 (91.7%) female
**Ethnicity**	23 Caucasian 3 African-Caribbean 4 Missing	12 Caucasian 3 African-Caribbean 1 Missing	9 Caucasian 3 Missing
**Mean no of previous admissions (standard deviation)**	1.1 (1.3)	1.5 (1.5)	0.4 (0.7)
**Mean no of palliative problems (standard deviation)**	4.1 (2.6)	5.1 (2.3)	2.8 (2.4)
**Mean no of inpatient multiprofessional disciplines seen this admission (standard deviation)**	1.6 (1.5)	2.1 (1.6)	0.9 (0.9)

### Ejection fraction and patient characteristics

Subsequent examination of ECHO data found 11 patients to have a confirmed ejection fraction ≤45%. Among these 11 patients with ECHO ≤45%, the mean patient age was 73.9 years (range 48–91), six were male, and nine were Caucasian. Their mean ejection fraction was 36.4% (SD = 6.7). They had a mean of 1.9 cardiac-related admissions in the previous 12 months (range 0–4). Prescribed medications were as follows: Loop diuretic n = 10, beta-blocker n = 6, aspirin n = 5, spironolatone n = 3, digoxin n = 3, ACE inhibitor n = 2. Among those 17 patients without ejection fraction ≤45%, prescribed medications were as follows: Loop diuretic n = 16, aspirin n = 9, ACE inhibitor n = 6, beta-blocker n = 5, spironolatone n = 3, digoxin n = 3.

### Appropriateness for palliative care

Of the 28 patients clinically identified on the wards as having CHF, 16 (57%) were identified as being appropriate for palliative care input, i.e. 4.4% of the inpatient population reviewed. Of the 11 with ejection fraction ≤45%, 10 (91%) were appropriate. Therefore, 11/365 (3.0%) of the entire inpatient population had clinical diagnosis of CHF and confirmed ejection fraction ≤45%, and of these 10 (2.7% of the inpatient population) were appropriate for palliative care.

On the date of the census, only one of the inpatients clinically identified as having CHF was currently known to the inpatient palliative care team.

### Ejection fraction and palliative care appropriateness

Eleven patients had both clinical diagnosis and confirmed ejection fraction ≤45%. A further 11 patients had chronic heart failure specified in their notes as a reason for their admission but had an ejection fraction of greater than 45%. Six were clinically identified as having CHF as a significant reason for admission by their ward medical staff during the census but had no ECHO data on file three months after the census date. Of the 17 patients with no supporting ECHO data (i.e. no ECHO result n = 6, or an ECHO result showing normal function n = 11), five (29.4%) were identified as being appropriate for palliative care.

### Characteristics of patients appropriate for palliative care

Those patients appropriate for palliative care had a mean of 5.1 unresolved symptoms and problems at 7 days post-admission.

The characteristics of the following two groups were compared to the remaining patients with a clinical CHF diagnosis: a) those identified as appropriate for palliative care irrespective of ECHO data, and b) those with ejection fraction ≤45% and palliative care appropriate.

Compared to the remaining patients with a clinical CHF diagnosis (n = 12), those identified as palliative care appropriate (n = 16) had a statistically significant higher mean number of previous admissions (1.53 compared to 0.44, p = 0.024, t = -2.433); were being seen by a significantly greater number of multiprofessional inpatient staff (i.e. 2.1 staff compared to 0.9, P = 0.045, T = -2.169), and were significantly more likely to have a "do not resuscitate order" in their notes (43.8% compared to 0%, p = 0.011, x^2 ^= 6.497).

Compared to all those remaining patients with a clinical diagnosis of CHF (n = 17), those with an ejection fraction ≤45% and appropriate for palliative care (n = 11) had a statistically significant higher mean number of previous admissions (1.9 compared to 0.57, p = 0.012, t = -2.733).

## Discussion

Given the challenges of decision-making regarding palliative care initiation for CHF patients due to movement between NYHA classification levels, the data describing characteristics associated with palliative care appropriateness is useful, particularly in the absence of ECHO data. The number of clinically identified CHF patients without ECHO data is indicative of the relevance of palliative care to all heart failure patients, including those elderly patients with normal systolic function, right sided heart failure and those with diastolic dysfunction.

### Limitations of the present study

This data is likely to report a conservative estimate of the point prevalence of CHF inpatients appropriate for palliative care, i.e. 2.7% after confirmed ECHO data. Firstly, the definition of being palliative care appropriate stated unresolved problems seven days post-admission, which may have excluded recent admissions who were actively dying, and those with recent admissions whose problems may have remained unresolved. However, the study aimed to measure problems unresolved after seven days under a cardiology admission, and which would likely benefit from referral to the specific palliative care team/specially trained cardiologists. Second, file recording of clinical diagnosis of CHF and symptom prevalence is likely to be lower than prevalence when prospectively assessed using a validated tool. This limitation is due to the methodological reliance on data recorded from routine clinical practice which may not adequately focus on the experience of pain and other symptoms. Third, the inclusion of a specific appraisal of family needs and communication needs (central concerns in the assessment and delivery of palliative care) are likely to have further increased the prevalence of unmet palliative care needs.

Further limitations in this study are that the hospital was a tertiary referral centre, although audit of CHF admissions showed that 90% of patients were resident in local Primary Care areas (unpublished data). It is also noteworthy that CHF patients tend to have a number of co-morbidities that may cause symptoms not related to CHF.

## Clinical consequences

This data, in conjunction with substantive data from the other components of the multi-methods study [10-12] informed the generation of evidence-based referral criteria to palliative care. However, CHF patients should remain under the care of cardiology teams where possible, who may offer generalist palliative care as appropriate. Specialist palliative care teams can offer consultation, co-management and care for complex cases, currently achieved in malignant disease care models. While palliative care aims to improve outcomes from the point of diagnosis, palliative care skills are needed for good management in advanced stages, and dialogue and support between palliative care and cardiology should inform when to refer and to what extent generalist palliative care skills can be provided by cardiac teams. [13]

The present data offer useful indications to cardiology teams of which patients may be appropriate for referral to palliative care. Those with a clinical diagnosis who were appropriate for palliative care had 1.53 admissions in the previous year and 5.1 unresolved current problems, were being seen by 2.1 multiprofessional non-medical non-nursing staff, and around half had a DNR order in their notes. Among those appropriate for palliative care with a confirmed CHF diagnosis using ECHO data, they had 1.9 previous admissions, and 5.8 problems.

Our data support the belief that CHF diagnosis is complex, and clinical suspicion needs to be supported by further investigation. [14] It is noteworthy that the majority of heart failure patients were identified as needing some palliative care input irrespective of ECHO data. This supports recent prospective clinical data demonstrating that patients with CHF and normal ejection fraction (i.e. >45%) have equally high levels of needs and similar survival rates as those with ejection fractions ≤45%. [15,16]This may be due to the high rate of co-morbidities among this relatively elderly population. Palliative care provision should be according to need.

Referral criteria and care pathways for this patient population need to take account of the complexities of prognostication and incidence of sudden death. [17] Palliative care planning that takes account of preferences and family support may reduce the number of unplanned admission among CHF patients (an internal audit [unpublished data] found that within the Hospital 22% of discharged heart failure patients were readmitted within 30 days).

## Conclusion

We propose referral criteria based on this data, mindful that referrals should not rely on end-of-life or terminal stages, as earlier intervention may optimise quality of life.

**Figure 2:**
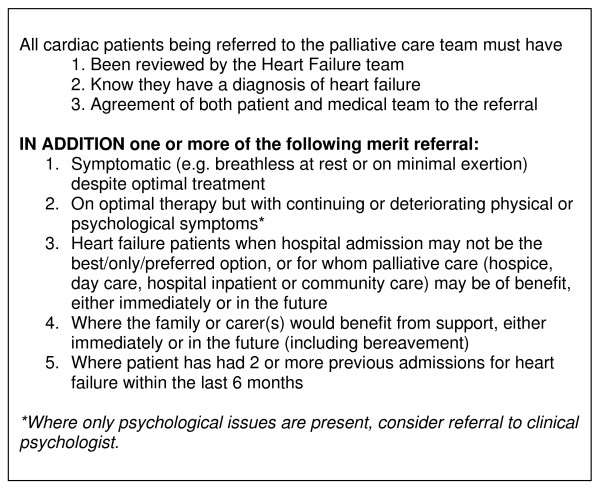
**Proposed referral criteria to palliative care for patients with Chronic Heart Failure**.

Our proposed criteria are reproduced in Figure 2.

Our conservative measurement of the magnitude of need suggests that 4.4% of medical, vascular surgical and care of the elderly hospital inpatients have clinically diagnosed CHF and require palliative care, therefore adequate generalist and specialist skills are required within the acute setting. We propose the present criteria as a means to ensure optimal quality of life for patients with CHF according to need rather than disease progression.

## Competing interests

The authors declare that they have no competing interests.

## Authors' contributions

RH designed the study, secured funding, managed data collection/analysis and drafted the manuscript. TB assisted design, secured funding, recruited subjects, assisted in interpretation and commented on drafts. FH was a member of the project group, recruited patients, assisted in interpretation and commented on drafts. EC was a member of the project group, recruited patients, assisted in interpretation and commented on drafts. MK was a member of the project group, participated in interpretation and commented on drafts. LS was a member of the project group, participated in interpretation and commented on drafts. IH was a member of the project group, assisted design, assisted in interpretation and commented on drafts. All authors read and approved the final manuscript.

## Pre-publication history

The pre-publication history for this paper can be accessed here:


